# Upper Back Pain: A Rare Clinical Presentation in Pericardial Effusion

**DOI:** 10.7759/cureus.20230

**Published:** 2021-12-07

**Authors:** Shikha Jha

**Affiliations:** 1 Internal Medicine, Saint Peter’s University Hospital, New Brunswick, USA

**Keywords:** cardiac tamponade, pericardiocentisis, atypical clinical, upper back pain, effusion

## Abstract

Pericardial effusion is an important cardiac condition seen in clinical practice with several known underlying etiologies. We are aware of the challenge that exists in diagnosing the effusion. An interesting challenge that has caught our attention is the highest chance of missing the diagnosis if there is an atypical clinical presentation of the patient. In this case report, our objective is to discuss a case that enhances the importance of careful and meticulous investigation of a patient with an atypical clinical picture. This is a case report of a 92-year-old woman who presented to the emergency department with a chief complaint of upper back pain for a few days. She was found to have cardiomegaly on further imaging. An echocardiogram showed a moderate size pericardial effusion. Pericardiocentesis was done and a drain was left in place. Of note, the patient reported remarkable resolution of the back pain after the fluid was taken out. The serial echocardiogram post pericardiocentesis showed minimal drainage, hence the drain was taken out, and the patient was observed for clinical monitoring 24 hours post drain removal. A timely diagnosis and treatment saved our patient from the most dreadful life-threatening condition along with a secure discharge from the hospital.

## Introduction

Pericardial effusion is the accumulation of excessive fluid in the pericardial sac around the heart. A normal pericardial sac in a healthy individual contains 15-50 mL of serous fluid, which may be exudative, transudative, or sanguineous [1]. The pericardial fluid may contain malignant cells or infectious organisms based on the underlying etiology. Some concerning etiologies are infection, inflammation, iatrogenic, traumatic injury, cardiac wall rupture, and ascending aortic dissection [2]. Pericardial effusion may be suspected from history, physical examination, electrocardiogram, and chest radiograph. Most patients without hemodynamically significant pericardial effusion will have no symptoms specific to the effusion but may present with symptoms related to the underlying cause [3]. Since pericardial effusion is possible across all ages and populations and the predominant etiology is variable, careful and thorough history, physical examination, and diagnostic approach are essential for patient care. There are not many studies done to show the association between upper back pain as presentation and pericardial effusion as underlying etiology. Especially, in the context of having very little data regarding the prevalence and incidence of atypical presentation in pericardial effusion, our case report emphasizes the importance of a clinician’s judgment in challenging situations. A timely diagnosis and treatment of the effusion can be a life-saving event for some of the most acute patient case scenarios.

## Case presentation

**Figure 1:**
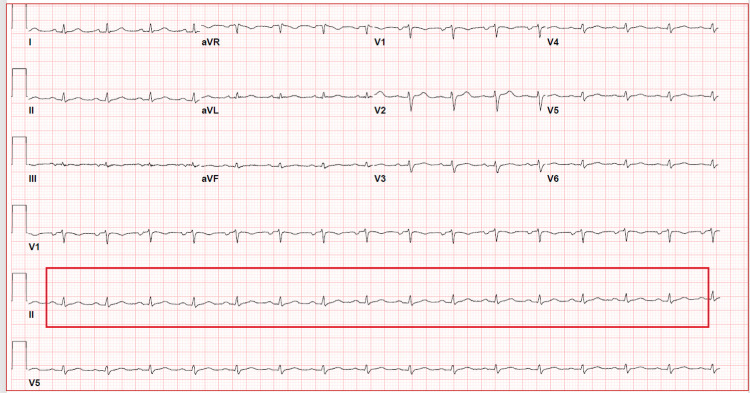
Electrocardiogram showing low voltage QRS

A 92-year-old female with a past medical history significant of hypertension, hypothyroidism, and gastroesophageal reflux disease (GERD) initially presented to the emergency department with the chief complaint of upper back pain. She reported that this back pain is acute in onset, has been ongoing for the past few days, associated with worsening fatigue and occasional palpitations. She has had chronic low back pain in the past but reports that the location and nature of this back pain are different from the past. No complaint of shortness of breath, chest pain, or palpitations. Vitals were stable and within normal limits. Physical examination was pertinent of muffled heart sounds, S1 and S2, on cardiac auscultation. There was no evidence of pulsus paradoxus, elevated jugular venous pressure, or Ewart sign. The back was kyphotic on inspection, with no evidence of spinal tenderness. The EKG was pertinent to low voltage QRS (Figure 1).

**Figure 2:**
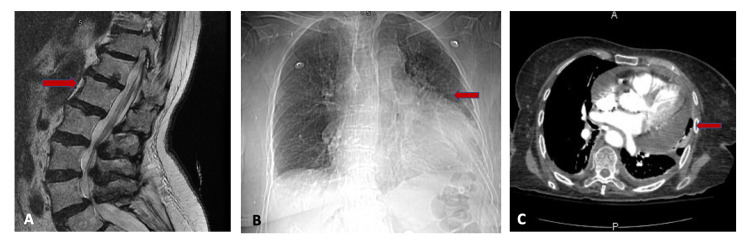
(A) MRI lumbar spine is showing degenerative and stenotic changes. CT pulmonary angiogram is showing cardiomegaly (B) and large pericardial effusion (C).

On review of the outpatient records, the patient had a recent MRI spine which showed degenerative and stenotic changes, with no evidence of mass effect or fracture (Figure 2A). The chest x-ray was pertinent for cardiomegaly and findings were suspicious of left base infiltrate. Given findings of infiltrates on the chest x-ray, the patient underwent CT pulmonary angiogram, which showed mild cardiomegaly (Figure 2B) and a large pericardial effusion (Figure 2C). Additionally, the imaging showed a 3 mm pulmonary nodule in the right upper lobe and 1.8 cm heterogeneous nodule anteriorly in the right thyroid lobe, grossly unchanged from the previous.

**Figure 3:**
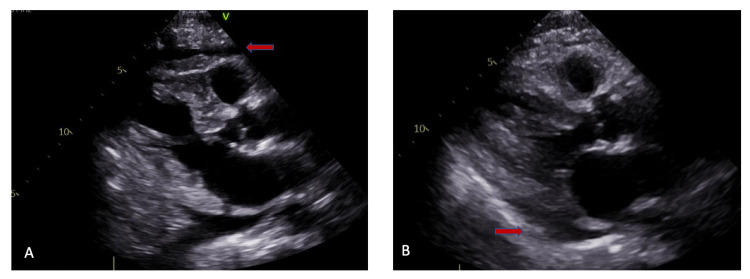
Two-dimensional (2D) echocardiography - (A) day 1 and (B) day 3 Day 1 - there is moderate circumferential pericardial effusion; day 3 - there is trace pericardial effusion.

In view of cardiomegaly, a two-dimensional (2D) echocardiography was done which showed a left ventricular ejection fraction of 60% with moderate, circumferential pericardial effusion, with no current evidence but indeed a high risk for cardiac tamponade (Figure 3A). The cardiology team was consulted who recommended pericardiocentesis. The patient underwent CT-guided pericardiocentesis, following which 300 cc bloody fluid was taken out with the drain being left in place [4]. On one hand, the patient was being worked up to find the underlying etiology of the pericardial effusion. On the other hand, the patient was being monitored for clinical improvement as he was in post-pericardiocentesis status with the drain in place [5,6]. Of note, the patient’s upper back pain remarkably improved after she underwent pericardiocentesis [7]. The subsequent drain value was 5 cc blood tinge fluid on the second day of the procedure. The second set of echocardiograms showed small, posterior-lateral pericardial effusion, following which the drain clamped for 24 hours, and then the third set of echocardiograms was done. The third echocardiogram showed only a trace amount of pericardial effusion with no fluid drainage 24 hours past the drain unclamping, following which the drain was taken out (Figure 3B).

The pericardial fluid analysis was done (Table 1). The fluid cytology was negative for malignant cells. There were reactive mesothelial cells and mixed inflammatory cells with abundant neutrophils. The QuantiFERON Gold test for tuberculosis was negative and the fluid adenosine deaminase (ADA) value was 65 units/L. Of note, her ANA came back to be positive with a titer of 1:102040. The other lab works were significant for hemoglobin of 10.2 g/dL and a mean corpuscular volume of 79 femtoliters. The values of thyroid-stimulating hormone and free T4 were 5.235 IU/mL and 1.72 ng/dL, respectively.

**Table 1:** Pericardial fluid analysis

Fluid type	Pericardial
Color	Red
Appearance	Cloudy
RBC	17,100/cubic mm
Total neutrophil count	1105/cubic mm
Poly%	74%
Lymph%	20%
Glucose	34 mg/dL
LDH	1881 Units/L
Protein	4 g/dL
Chloride	104 mmol/L
Adenosine deaminase	64.3 Units/L
Gram stain	Many polymorphonuclear leucocytes
Culture	No growth

Hence, over the course of three days, in the view of hemodynamic, clinical, and radiological improvement, the patient was discharged to home with a recommendation to have outpatient follow-up with her primary medical doctor for further medical management, follow-up with cardiologist for further monitoring of the cardiac condition. She was also strongly recommended to see a rheumatologist for a further autoimmune workup to determine the underlying etiology. Also, given the evidence of right upper lobe pulmonary nodule, the patient was set up with the pulmonary team for outpatient follow-up, as malignancy is also one of the most potent causes of pericardial effusion [8].

## Discussion

Pericardial effusion can apparently occur in any condition that impacts the pericardium. Pericardial effusion presentation varies from person to person depending on the size, acuity, and underlying cause of the effusion [9]. Some of the most common causes are acute pericarditis from the viral, bacterial, tubercular, or idiopathic origin, autoimmune conditions, cardiac injury or infarction, chest trauma, radiation, malignancy, uremia, myxedema, medication. Some people may be asymptomatic and the effusion may be an incidental finding on an examination [10]. Larger effusions may present with chest pressure or pain, dyspnea, shortness of breath, and malaise. Patients with cardiac tamponade, a life-threatening complication, may present with low blood pressure, restlessness, hyperventilation, discomfort with laying flat, dizziness, and syncope [11]. Noncardiac symptoms may also present due to the enlarging pericardial effusion compressing nearby structures, such as nausea and abdominal fullness, dysphagia, hiccups, due to compression of stomach, esophagus, and phrenic nerve respectively [12]. However, in the absence of cardiac tamponade, signs, and symptoms might not be sensitive or specific for pericardial effusion. Unless cardiac tamponade is present, the clinical presentation is so insensitive and nonspecific that they are of historic rather than practical interest, for instance, the presentation in our patient case as mentioned above. Most patients without a hemodynamically significant pericardial effusion will have no symptoms specific to the effusion. The pericardial effusions are often discovered incidentally during diagnostic evaluation. The diagnostic approach consists of the following three steps: confirming the presence of pericardial effusion; assessing its hemodynamic impact, if any; and, whenever possible, establishing the cause of the pericardial effusion. A transthoracic echocardiogram is usually sufficient to evaluate pericardial effusion and it may also help distinguish pericardial effusion from pleural effusion and MI. Most pericardial effusions appear as an anechoic area (black or without an echo) between the visceral and the parietal membrane. Complex or malignant effusions are more heterogeneous in appearance, meaning they may have variations in echo on ultrasound [9]. Pericardiocentesis is a procedure used to analyze the fluid but more importantly can also provide symptomatic relief, especially in patients with hemodynamic compromise. The drainage tube is often left in place for 24 hours or more for assessment of re-accumulation of fluid and also for continued drainage [12].

## Conclusions

The core reason for presenting the above case report is to enhance awareness among clinicians, about the clinical picture completely nonspecific to pericardial effusion that a patient may present with. In such exceptional circumstances, careful and thorough investigations aid a clinician to diagnose pericardial effusion at the right time, leading to timely intervention. Hence, timely procedural intervention can save our patients from the most life-threatening conditions such as tamponade. After the acute procedural management of the effusion, it is equally important to ensure that the patients have close and continuous monitoring with expertise at the ambulatory level to watch for effusion recurrence or progression via interval echocardiogram. A detailed inpatient, as well as ambulatory level, follow-up for laboratory and imaging workup to identify the underlying etiology for the pericardiac effusion, is crucial.
